# Influence of Zirconia Type and Surface Treatment on the Shear Bond Strength Between Veneering Porcelain and Zirconia

**DOI:** 10.3390/dj14090586

**Published:** 2026-09-11

**Authors:** Kamolporn Wattanasirmkit, Karnkanith Hansomwong, Trakol Mekayarajjananonth, Sunphat Namano, Jaijam Suwanwela, Taksid Charasseangpaisarn

**Affiliations:** 1 Department of Prosthodontics, Faculty of Dentistry, Chulalongkorn University, Bangkok 10330, Thailand; 2 Department of Prosthodontics, College of Dental Medicine, Rangsit University, 52/347 Mueang-Ake, Lak Hok, Pathum Thani 12000, Thailand

**Keywords:** shear bond strength, surface roughness, surface treatment, translucent zirconia, veneering porcelain, zirconium oxide

## Abstract

**Background:** Proximal contact loss is a major problem in zirconia restorations on implant abutments. This study investigated the effect of surface treatments on the shear bond strength (SBS) between veneering porcelain and translucent zirconia (4Y-PSZ and 5Y-PSZ) compared with conventional zirconia (3Y-TZP). **Methods:** Forty-four specimens of each zirconia type (10.0 × 7.0 × 1.0 mm) were prepared and randomly assigned to four surface treatment groups: control (C), grinding with 150-grit silicon carbide paper (GR), airborne-particle abrasion with alumina particles (AB), and glazing (GL). Surface roughness (Ra) was measured for one specimen from each treatment group. Veneering porcelain cylinders (2.38 mm in diameter, 0.5 mm in thickness) were fabricated and bonded to the zirconia surfaces. SBS was evaluated using a universal testing machine (*n* = 10). Data were analyzed using two-way ANOVA and Tukey’s HSD test (α = 0.05). **Results:** The GL group exhibited the lowest Ra, whereas the AB group showed the highest Ra. Regarding zirconia type, 3Y-TZP demonstrated significantly higher SBS than 5Y-PSZ, while no significant differences were found between 3Y-TZP and 4Y-PSZ or between 4Y-PSZ and 5Y-PSZ. The AB and GL groups showed significantly higher SBS than the C group, whereas the GR group did not differ significantly from the C group. **Conclusions:** Airborne-particle abrasion and glazing effectively enhanced SBS across the zirconia types evaluated prior to veneering.

## 1. Introduction

Monolithic zirconia full anatomic contour crowns are widely used in implant-supported restorations due to their survival and have success rates of more than 90% [1,2,3]. Zirconia is a polymorphic metastable material that has three crystallographic phases: monoclinic, tetragonal, and cubic. Stabilized oxides are added to zirconia to prevent phase transformation, resulting in stabilized zirconia at room temperature. To stabilize tetragonal zirconia, 3 mol% yttrium oxide (Y_2_O_3_) is used, which is called 3 mol% yttria-tetragonal zirconia polycrystal (3Y-TZP). 3Y-TZP is used in prosthetic dentistry for dental crowns and fixed partial denture (FPD) substructures with veneering porcelain. However, the major problem with veneering zirconia is the chipping of the veneering porcelain, with a prevalence of 15.2% after five years [4]. The bond strength between zirconia and porcelain is influenced by various factors, such as differences in the coefficients of thermal expansion (CTEs) between materials [5,6,7], thermal conductivity [8,9], defects at the interface [10], and veneering porcelain thickness [11].

Monolithic translucent zirconia for full anatomic contour crowns has been developed to enhance their optical characteristics and to solve issues related to veneering porcelain debonding. Monolithic translucent zirconia is a partially stabilized zirconia (PSZ) that contains a higher amount of yttria stabilizer than conventional zirconia; hence, it contains a higher amount of cubic phase. Specifically, zirconia with 4 mol% yttria is called 4Y-PSZ, while that with 5 mol% yttria is referred to as 5Y-PSZ [12,13]. After the zirconia restoration is fabricated, it can be either glazed or polished to smoothen the surface before delivering the prosthesis [14].

Proximal contact loss (PCL) between an implant prosthesis and the adjacent natural tooth is a common complication after implant prosthesis delivery [15]. PCL rates are reported to be 59.9% within 3 years [16]. The amount of PCL is 0.33 ± 0.14 mm in posterior implant restorations, with patients reporting food impaction [17]. The management of PCL aims to recreate the optimal proximal contact; however, the decision to repair or replace the existing restoration depends on several factors, such as material retrievability, finances, esthetics, and width of the proximal gap [18].

Zirconia implant-supported prostheses with an open proximal contact can be modified by adding either veneering porcelain or resin composite [19,20]. There are few reports concerning repairing porcelain on monolithic translucent zirconia substructures. Moreover, one in vitro study reported that the shear bond strength between porcelain and monolithic 6Y-PSZ was lower than that of 3Y-TZP [21]. Previous studies showed that grinding and airborne-particle abrasion on the zirconia surface are methods used to enhance the bond strength between the substructure and veneering material. Grinding with a diamond bur or airborne-particle abrasion with aluminum oxide (Al_2_O_3_) increases the surface area, surface roughness and provides undercuts, which increases the mechanical interlocking between the zirconia and veneering porcelain [22,23,24,25,26].

After the mechanical surface treatment of zirconia, the phase on the surface can be changed from tetragonal to monoclinic [27]. Additionally, the rhombohedral phase, a distorted form of the cubic and tetragonal phases, is observed in surface-treated, partially and fully stabilized zirconia. Thus, heat treatment has been recommended after mechanical surface treatment procedures for reversing the monoclinic phase to the tetragonal phase, and the relative content of the rhombohedral phase also decreases after heat treatment [28,29,30]. However, heat treatment of zirconia following airborne-particle abrasion can significantly decrease the bond strength and overall strength of zirconia [31,32].

The purpose of the present study was to evaluate the effect of different zirconia surface treatments on shear bond strength (SBS) between veneering porcelain and translucent zirconia (4Y-PSZ and 5Y-PSZ) compared with conventional zirconia (3Y-TZP). The null hypotheses tested in this study were that the type of zirconia, with each containing a different mol% Y_2_O_3_ as a stabilizer, would have no effect on the SBS between veneering porcelain and zirconia, and the different methods of surface treatment on zirconia would have no effect on the SBS between veneering porcelain and zirconia.

## 2. Materials and Methods

### 2.1. Zirconia Specimen Preparation

Forty-four specimens of 3Y-TZP, 4Y-PSZ and 5Y-PSZ were prepared from zirconia disks with dimensions of 10.0 × 7.0 × 1.0 mm (Table 1) and were sintered according to the manufacturer’s recommendations (Table 2). The specimens were ultrasonically cleaned in distilled water for 10 min with ultrasonic cleaner (Bransonic model 5210, Branson, Danbury, CT, USA) and thoroughly dried with compressed air.

### 2.2. Surface Treatment

The specimens were labeled with an individual number and randomly allocated to 4 subgroups using a computer-generated random sequence (*n* = 11). The different surface treatments were applied to the top surface of the specimens according to the surface treatment protocol (Table 3). After that, the specimens were ultrasonically cleaned in distilled water for 10 min. Regeneration firing as recommended by the manufacturer was performed after surface treatment for the C, GR and AB groups. For the GL group, regeneration firing was done before applying porcelain (Table 2).

### 2.3. Phase Identification of Zirconia

One specimen from the C, GR, and AB groups was randomly selected for phase identification. Monoclinic, tetragonal, cubic, and rhombohedral phase identification and quantitative analysis of zirconia phases from each group except the glazing group were performed. The process was done by X-ray diffraction (D8 Discover; Bruker AXS GmbH, Karlsruhe, Germany), using Cu-Kα radiation at 40 kV and 40 mA. The XRD patterns were collected from 20 to 90° with a step size of 0.02°. The XRD pattern was compared with the diffraction standards of the International Center for Diffraction Data (ICCD or JCPDS). The phase quantity was calculated using Topas Software (TOPAS-Academic Version 6; Coelho software, Brisbane, Australia).

### 2.4. Surface Roughness Measurement and Morphology

Following phase identification, the same specimen was used for surface roughness measurement in the C, GR, and AB groups. For the GL group, which was not subjected to phase identification, one specimen was randomly selected for surface roughness measurement only. The surface roughness (Ra) of zirconia before and after surface treatment was analyzed using a contact optical profilometer (Talyscan 150; Taylor Hobson, Leicester, UK). The difference in Ra was calculated and recorded as representative for each group. The surface morphology was also observed under a stereomicroscope (SZ61; Olympus, Tokyo, Japan) at 40× magnification.

### 2.5. Zirconia and Veneering Porcelain Specimen Preparation

**Figure 1:**
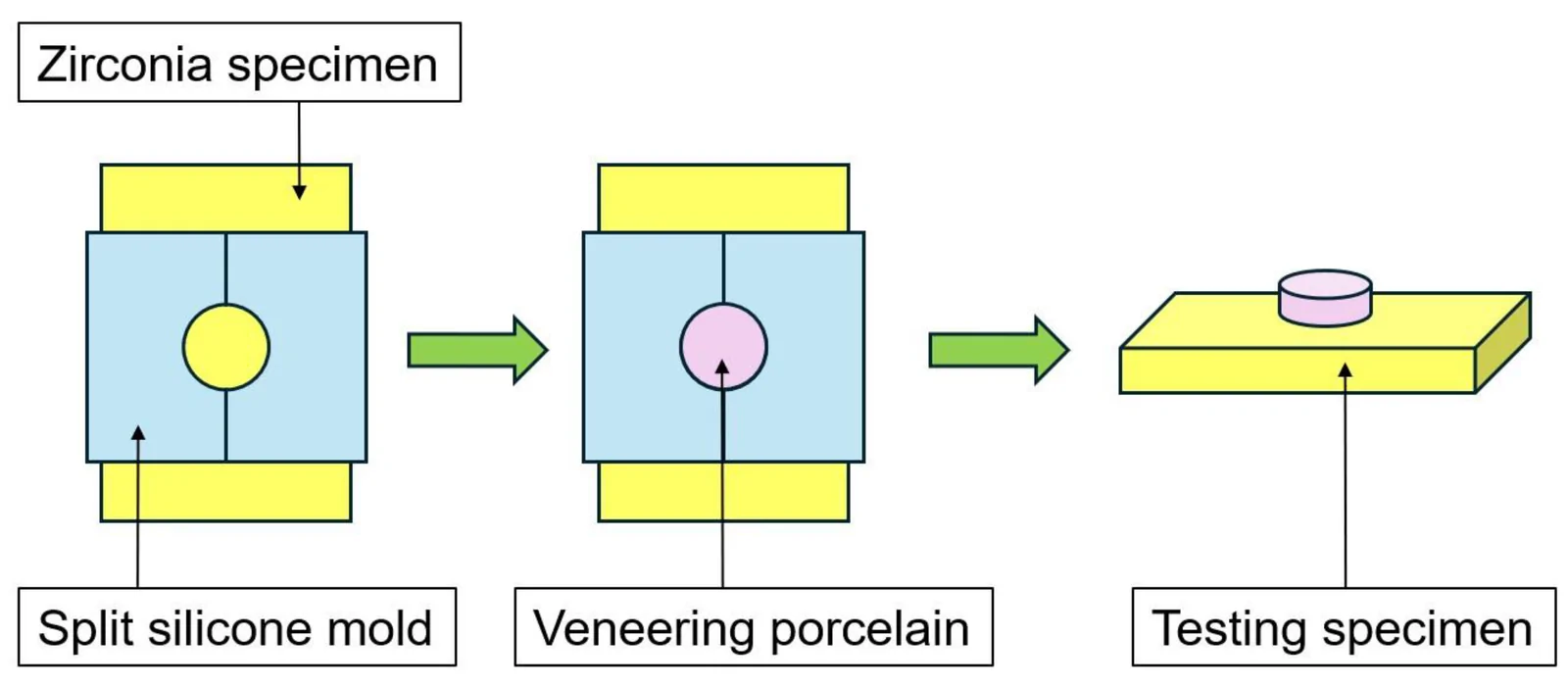
Preparation of the bonded zirconia (yellow) and veneering porcelain (pink) specimen.

Porcelain cylinders were fabricated with a custom-made split silicone mold. Porcelain powder was mixed with the appropriate amount of modeling liquid and condensed to form a cylinder 2.38 mm in diameter and 0.5 mm in thickness. Excess water was blotted with tissue paper. Firing was performed in a porcelain furnace (Programat P300; Ivoclar Vivadent, Liechtenstein, Austria) per the manufacturer’s instructions (Table 2). The process is shown in Figure 1.

### 2.6. Shear Bond Strength (SBS) Test

The remaining ten specimens of each group were subjected to an SBS test (*n* = 10). The porcelain veneering bonded zirconia specimens were embedded in a polyvinyl chloride ring using polymethyl methacrylate (PMMA) resin and stored in distilled water at 37 °C for 24 h prior to testing. The tests were performed using a universal testing machine (EZ-S; Shimadzu, Kyoto, Japan) according to ISO 29022:2013 [33]. A shear load was applied at a crosshead speed of 1 mm/min until failure occurred. The ultimate load at failure was recorded; the average shear bond strength (MPa) was calculated from the following formula: SBS (MPa) = Load (N)/area (mm^2^). The mean shear bond strength and the standard deviation for each specimen group were calculated.

### 2.7. Failure Analysis

The failure analysis of each specimen was performed under a stereomicroscope at 20× magnification. The failure modes were classified as adhesive, cohesive and mixed failure. Adhesive failure occurred between the interfaces of the zirconia and veneering porcelain. Cohesive failure occurred in either the zirconia or veneering porcelain. Mixed failure was a combination of adhesive and cohesive failures. The specimen identification numbers were masked with opaque paper prior to failure-mode assessment to blind the examiner to specimen identity.

One sample from each group was randomly selected. The specimens were coated with gold using sputter-coating (JFC-1200E Fine coater; JEOL Ltd., Tokyo, Japan). The zirconia–porcelain interface and fractured surface were observed by SEM at 80× and 2000× magnification.

### 2.8. Statistical Analysis

The sample size for the present study was estimated based on the effect size reported in a previous study [23]. A priori power analysis was performed using G*Power (Version 3.1.9.7, G-Power, Düsseldorf, Germany) for a two-way ANOVA (fixed effects, special, main effects and interactions), with a numerator df of 6 and 12 groups, an α level of 0.05, a statistical power of 80%, and an effect size of f = 0.38. The calculated minimum total sample size (*N*) was 102 specimens. The present study therefore included 10 specimens per group (*n* = 10), for a total of 120 specimens, providing adequate statistical power to achieve the predetermined significance level.

The data were analyzed using IBM SPSS Statistics for Windows (Version 22.0; IBM, Armonk, NY, USA). Two-way ANOVA was used to determine the effects of the types of zirconia and surface treatment methods and their interaction on the SBS. Post hoc comparisons of each factor were performed using Tukey’s honest significant difference (HSD) test at the 95% confidence level.

## 3. Results

### 3.1. XRD Analysis

The phase contents of the different types of zirconia and phase transformation after GR and AB are presented in Table 4. The rhombohedral phase increased after grinding and airborne-particle abrasion with Al_2_O_3_ in all types of zirconia. In the control group, the higher the mol% of Y_2_O_3_, the greater the wt% of the cubic phase of zirconia.

### 3.2. Surface Roughness and Morphology

**Figure 2:**
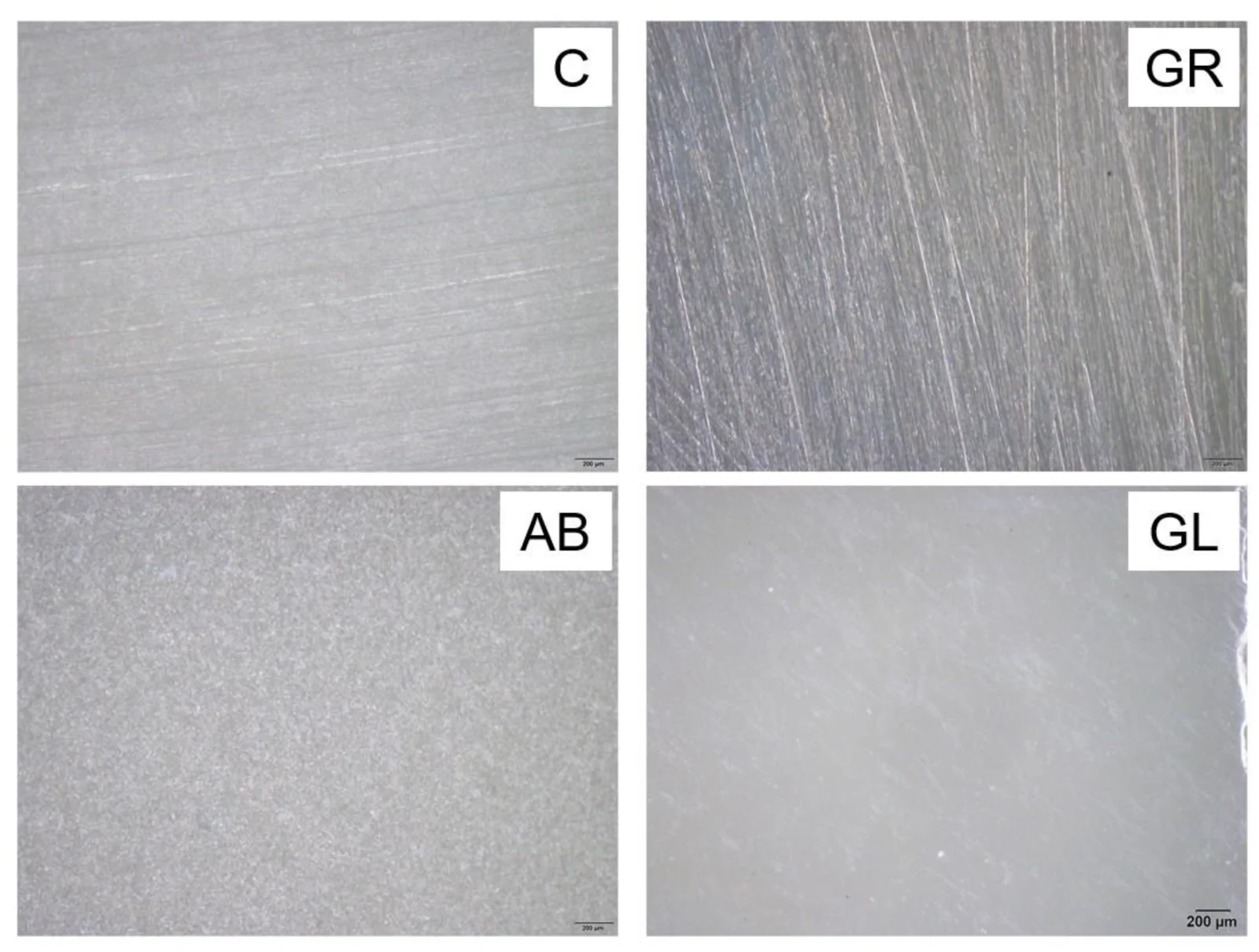
Surface morphologies of zirconia types after surface treatment: control (C), grinding (GR), airborne-particle abrasion (AB), glazing (GL).

The Ra values of the different types of zirconia are shown in Table 5. The AB group had the highest surface roughness (Ra) compared with the other surface treatments, followed by C, GR and GL. The surface morphologies of the zirconia substructures after surface treatment at 40× magnification are presented in Figure 2. The surface morphology demonstrated different patterns of roughness for each surface treatment. Grinding produced surface morphologies with varying roughness levels, which are presented in the same direction as the grinding strokes. In the AB group, the surface morphology was shaped by the abrasive action, resulting in the formation of pits and mounds of varying depths. In the GL group, the specimen presented a smooth surface formed by the glazed porcelain.

### 3.3. Shear Bond Strength Test and Mode of Failure

**Figure 3:**
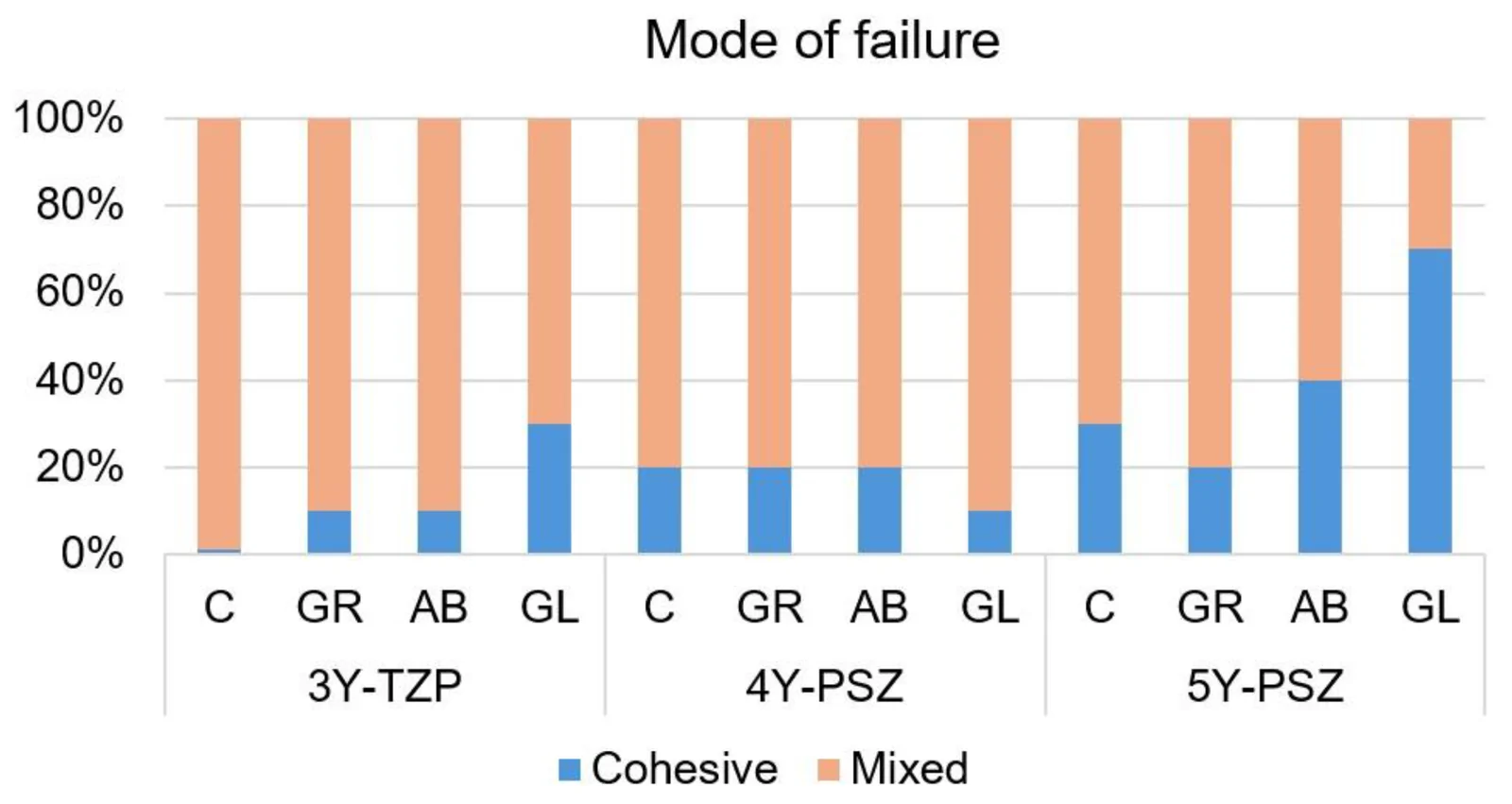
The prevalence of each mode of failure in each group.

The one-sample Kolmogorov–Smirnov test showed a normal distribution of SBS values within each group. The two-way ANOVA showed that the main effect of type of zirconia (F(2,108) = 10.02, *p* < 0.001, partial η^2^ = 0.157, 95% CI [0.045, 0.276]) and surface treatment (F(3,108) = 8.52, *p* < 0.001, partial η^2^ = 0.191, 95% CI [0.063, 0.309]) was significantly different (*p* < 0.001), whereas the interaction between two factors (F(6,108) = 1.47, *p* = 0.196, partial η^2^ = 0.075, 95% CI [0.000, 0.144]) was not significantly different, as shown in Table 6. The types of zirconia and surface treatment were therefore analyzed separately, and Tukey’s HSD test was used to determine the differences between groups within each factor. The results of the SBS analysis are shown in Table 7, and the mode of failure for each group is shown in Figure 3.

**Figure 4:**
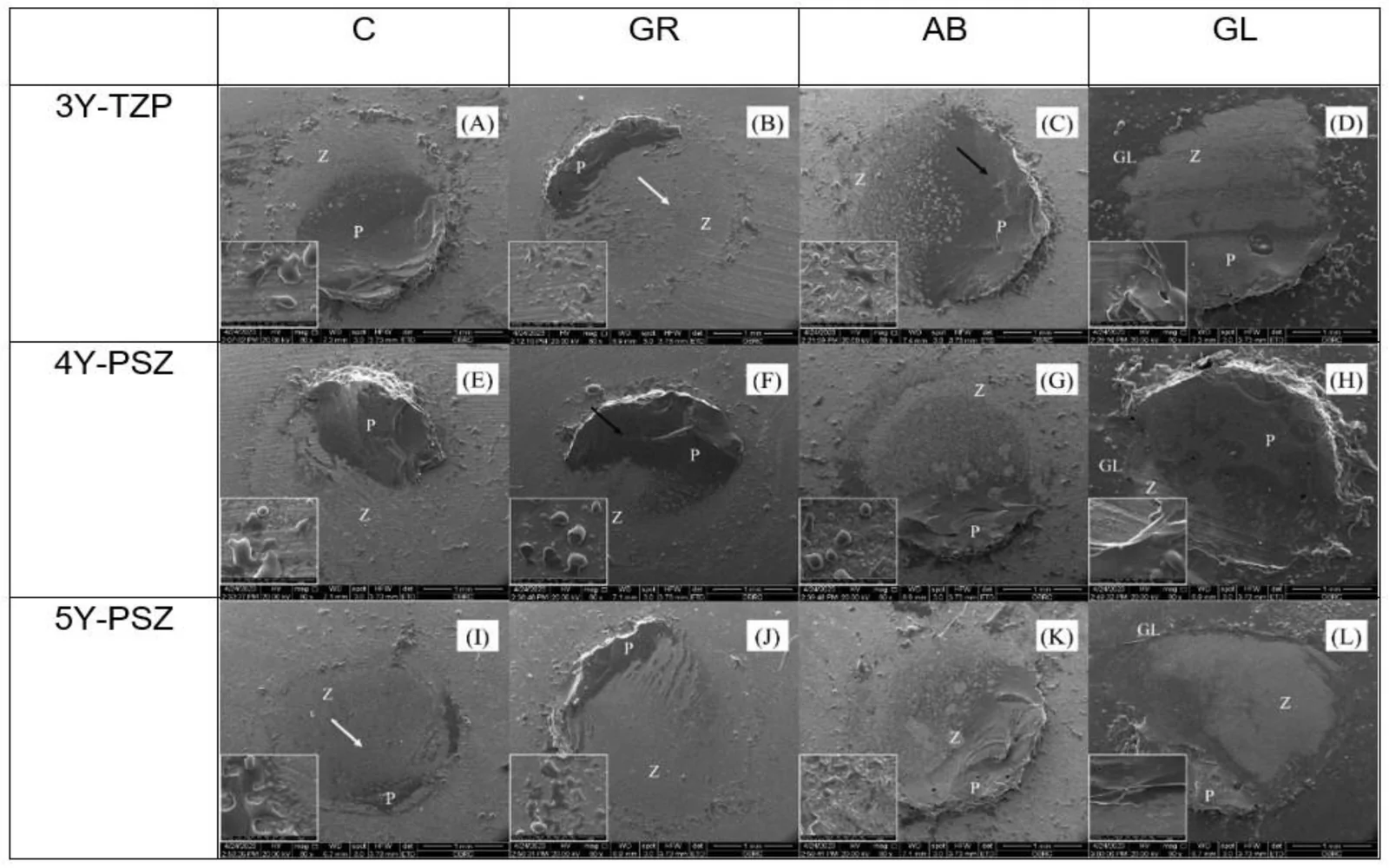
SEM images of the fracture surface at 80× magnification and 2000× magnification are presented in the lower left corner. Z indicates an exposed zirconia surface; P indicates a fractured porcelain surface; and GL indicates glazed porcelain. White arrows in (**B**,**I**) indicate adhesive failure; black arrows in (**C**,**F**) indicate cohesive failure within the porcelain layer.

The SEM images of the fracture surface at 80× and 2000× magnification are presented in Figure 4. In mixed failure, fractures originated at the veneering porcelain layer and deflected through the veneering porcelain and zirconia core interface.

## 4. Discussion

In the present study, the null hypotheses were rejected, as the type of zirconia and surface treatment affected the shear bond strength between veneering porcelain and zirconia. The veneering porcelain on the 3Y-TZP substructure groups exhibited the highest bond strength, followed by 4Y-PSZ and 5Y-PSZ.

The majority of failures in this study were mixed failures with some cohesive failure in veneering porcelain. The majority of the fractures originated at the porcelain layer and deflected through the veneer–core interface, which indicated that the bond strength between zirconia and veneering porcelain was lower than the porcelain strength. This finding is consistent with previous studies in which the SBS values were close to those obtained in this study, and had a majority mixed failure mode whereby small remnants of veneering porcelain attached to the zirconia substrate [34,35]. This was in contrast to some previous studies, which showed that the predominant adhesive failures were between veneering porcelain and zirconia substrate or cohesive failure in veneering porcelain [36,37].

The bond strength between zirconia and veneering porcelain was influenced by multiple factors, including CTE mismatch, types of zirconia, and surface treatment methods. The mismatch in CTEs between the substructure and veneering porcelain produces both compressive and tensile residual stresses at the interface that affect the bond strength of these materials. A zirconia substructure with a slightly higher CTE leads to acceptable residual compressive stress in the porcelain layer [5,38]. The tensile stress in the porcelain layer causes crack propagation parallel to the interface [7]. Spontaneous delamination of porcelain occurred when the CTE mismatch value was 2.0 × 10^−6^ K^−1^ [39]. Thus, spontaneous delamination of the veneering porcelain was not observed in this study. The CTE mismatch of zirconia and veneering porcelain used in the present study was 1.3–1.5 × 10^−6^ K^−1^ (3Y-TZP), 1.1–1.3 × 10^−6^ K^−1^ (4Y-PSZ) and 0.8–1.0 × 10^−6^ K^−1^ (5Y-PSZ). The result for the control group in this study found that the 3Y-TZP group showed a significantly higher SBS value than 5Y-PSZ, but it was not significantly different from that of 4Y-PSZ, while SBS between the 4Y-PSZ and 5Y-PSZ groups was not significantly different. This finding is consistent with the studies of Saito et al. and Guess et al., who reported that CTE mismatch of zirconia substructures and veneering porcelain in the ranges of 1.0–1.7 × 10^−6^ K^−1^ and 0.75–1.7 × 10^−6^ K^−1^ did not affect the SBS value [5,8]. Furthermore, the study of Dimitriadis et al. also found that the bond strength of veneering porcelain on 3Y-TZP was higher than that on 4Y-PSZ in a three-point bending test [40]. However, this is contrasted by the study of Juntavee and Dangsuwan, who found that a CTE mismatch of 0.77–0.87 × 10^−6^ K^−1^ could provide higher SBS than one of 0.94 × 10^−6^ K^−1^ [39]. This might indicate that while CTE mismatch was observed between the types of zirconia, this might not be the main factor that affected the SBS value.

The different types of zirconia contain different amounts of tetragonal and cubic phases that affect many characteristics. First, 3Y-TZP contains a higher amount of tetragonal phase that could change to monoclinic phase when force is applied, called ‘transformation toughening’, leading to an increase in the strength of the zirconia and the bond strength. Second, the tetragonal phase has higher surface reactivity than the cubic phase, which causes higher chemical reactivity on the surface. Lastly, the grain size of translucent zirconia is larger than that of conventional zirconia due to lower Al_2_O_3_ content. The smaller grain size exhibited higher interfacial free energy. These phase-related surface characteristics are intrinsic material properties of zirconia that may influence bonding through mechanical interlocking and surface reactivity regardless of the veneering material; while direct evidence for this mechanism has primarily been reported in adhesive-resin bonding studies [41], a similar phase-dependent influence on bonded veneering porcelain is possible but has not yet been directly confirmed.

From previous studies, thermal conductivity also affects bond strength between zirconia and veneering porcelain. The metal alloys used in dentistry have high thermal conductivity, ranging from 40 to 300 Wm^−1^ K^−1^, while zirconia substructures exhibit values of 2.0–2.2 Wm^−1^ K^−1^. The thermal conductivity for felspathic porcelain is close to that of the zirconia substructure. During the cooling process, the low thermal conductivity of zirconia can generate residual stress in the porcelain layer, resulting in lower SBS values [8,9]. Each phase composition of zirconia also leads to different thermal conductivity. According to Song et al., the cubic phase demonstrated lower thermal conductivity than the monoclinic and tetragonal phases [42]. In this study, the XRD analysis revealed that 5Y-PSZ had a higher cubic phase content (wt%) than 4Y-PSZ and 3Y-TZP. Therefore, the lower SBS of 5Y-PSZ could be attributed to the poor thermal conductivity of the cubic phase.

The XRD analysis indicated that mechanical surface treatment with grinding and airborne-particle abrasion with Al_2_O_3_ increased the monoclinic and rhombohedral phase. The amount of 20–30% phase transformation from cubic to rhombohedral phase was observed in all types of zirconia after grinding and airborne-particle abrasion. This was also observed by Wertz et al., who found that the 3Y-TZP, 4Y-PSZ and 5Y-PSZ phases could be transformed to a rhombohedral phase after coarse polishing [43]. After the heat treatment process, zirconia transforms its phase from the monoclinic and rhombohedral phases to a tetragonal phase [30]. Our study followed the heat treatment protocol as recommended by the manufacturer. However, not all monoclinic and rhombohedral phases transform into tetragonal phases [29]. Grigore et al. found that the monoclinic phase content of mechanical surface-treated zirconia fell below 1% after veneering thermal treatment; this differed from the present study, which found that the monoclinic phase content fell by about 2.8–3.9% [28]. These different phases of zirconia also exhibit different CTEs. For example, the monoclinic phase has a CTE of 7.5 × 10^−6^ K^−1^ and the tetragonal phase has a CTE of 10.8 × 10^−6^ K^−1^. The CTE mismatch leads to tensile stress in the veneering porcelain and affects the strength of the bond between the veneering porcelain and zirconia substructure [26].

In the present study, 4Y-PSZ and 5Y-PSZ showed lower initial roughness. This might be due to a higher content of cubic phases, which are larger and more stable than the tetragonal phase in 3Y-TZP. The surface morphology of each group was changed after the surface treatments. However, based on the descriptive Ra values, no apparent relationship was observed between surface roughness and the SBS value. The surface treatment by grinding produced obvious long, non-uniform linear grooves with a V- or U-shaped profile when compared to the control group, but did not significantly increase the SBS when compared to control group. This observed morphology also did not align with the Ra value, which was lower for grinding than for the control group. This phenomenon might be attributed to the fact that Ra represents the arithmetic means of vertical surface deviations rather than the visual prominence of surface features. However, grinding enhanced the SBS of the 4Y-PSZ and 5Y-PSZ substructures above the minimum bond strength that was required, indicating the usefulness of this surface treatment.

The alumina sandblasting created random, uniform, crater-like pits distributed across the zirconia surface. The airborne-particle abrasion with Al_2_O_3_ and glazing of the zirconia surface increased the SBS between zirconia and veneering porcelain when compared with the control group. These results are consistent with previous studies that indicated that airborne-particle abrasion with Al_2_O_3_ improved the bond strength between 3Y-TZP and veneering porcelain [10,25]. Kutsuma et al., reported that the SBS of veneering porcelain with 3Y-TZP was higher than that with 6Y-PSZ. The application of airborne-particle abrasion with Al_2_O_3_ also enhanced the SBS in 3Y-TZP and 6Y-PSZ [21]. However, several studies found that this treatment did not significantly improve the bond strength in both 3Y-TZP [26,31] and 4Y-PSZ [44], which is different from the result of this study.

In one study, the result of glazing showed that the SBS values could be increased when compared to the control. The surface morphology of glazing showed the smoothest surface with the lowest Ra value among groups. This might be due to the high glass phase content and fine particle size. The glazing was able to flow and fill in the irregularities of the zirconia substructure better than veneering porcelain alone. Then the glazing could be bonded to the veneering porcelain directly [45]. Corresponding to our results, applying glazing on 4Y-PSZ and 5Y-PSZ prior to adding veneering porcelain resulted in higher SBS than the control group.

From the results of this study, we can conclude that when the contact of the zirconia restoration is loose and glazing still exists in the contact area, the veneering porcelain can be directly added. However, if glazing does not exist, the surface should be treated by airborne-particle abrasion prior to adding the veneering porcelain. This procedure may therefore be considered broadly applicable across the zirconia types evaluated in this study, including 3Y-TZP, 4Y-PSZ, and 5Y-PSZ.

Limitations of this study should be acknowledged. First, bond strength was evaluated under non-aged conditions; the absence of thermal, mechanical, and chemical aging (e.g., thermocycling, cyclic loading, storage in simulated saliva) means the reported SBS values may not directly reflect long-term intraoral performance. Second, the flat zirconia specimens bonded to cylindrical veneering porcelain specimens, tested following ISO 29022, may not represent the curved clinical contact geometry of a proximal contact repair. Thus, the effect of contour geometry on bond strength should be investigated in future studies. Moreover, further studies simulating the intraoral use of zirconia restorations are warranted. These should include evaluating the effect of thermocycling on tensile or microtensile bond strength between porcelain and zirconia veneers, using different testing methods to confirm the present findings, and assessing the effect of different immersion solutions simulating the oral environment and the effect of surface treatments on multi-composition zirconia.

The surface roughness and XRD analysis in this study were evaluated using only one specimen per group; these data should therefore be interpreted as descriptive rather than statistically generalizable. Future studies should include a larger number of specimens per group to permit statistical comparison among treatment groups and provide a more reliable estimate of variability. Atomic force microscopy (AFM) is recommended for further study to obtain higher resolution and quantitative 3-D topography of the treated surface.

## 5. Conclusions

Given the limitations of this in vitro study, it can be concluded that when zirconia is selected and the translucency of zirconia is not a primary consideration, 3Y-TZP or 4Y-PSZ outperform 5Y-PSZ due to their higher bond strength with veneering porcelain.

In cases of proximal contact loss, the zirconia surface should be modified prior to veneering porcelain application to increase bonding between porcelain and zirconia. Airborne-particle abrasion with Al_2_O_3_ and glazing produced significantly higher SBS than the control group. Grinding alone did not significantly improve bond strength compared with the control group and therefore may not be an effective surface treatment for enhancing zirconia–veneering porcelain bonding under the conditions of this study. These surface treatments, airborne-particle abrasion and glazing, demonstrated higher SBS under the experimental conditions of the present study and may be considered promising approaches for clinical zirconia repair, subject to validation in aged and clinically simulated conditions.

## Figures and Tables

**Table 1:** Details of the materials used in this study.

Materials		Manufacturer	Batch No.	Main Composition (wt%)	CTE (10^−6^ K^−1^)
Zirconia	3Y-TZP VITA YZ^®^ HT	VITA Zahnfabrik, Bad Säckingen, Germany	76260	ZrO_2_ (90–95%), Y_2_O_3_ (4–6%), HfO_2_ (1–3%), Al_2_O_3_ (0–1%), Pigments (0–1%)	10.5
	4Y-PSZ VITA YZ^®^ ST	VITA Zahnfabrik, Bad Säckingen, Germany	94390	ZrO_2_ (88–93%), Y_2_O_3_ (6–8%), HfO_2_ (1–3%), Al_2_O_3_ (0–1%), Pigments (0–1%)	10.3
	5Y-PSZ VITA YZ^®^ XT	VITA Zahnfabrik, Bad Säckingen, Germany	75410	ZrO_2_ (86–91%), Y_2_O_3_ (8–10%), HfO_2_ (1–3%), Al_2_O_3_ (0–1%), Pigments (0–1%)	10.0
Veneering Porcelain	VITAVM^®^9 Corrective	VITA Zahnfabrik, Bad Säckingen, Germany	75920	SiO_2_ (60–64%), Al_2_O_3_ (13–15%), K_2_O (7–10%), Na_2_O (4–6%), B_2_O_3_(3.3–5%)	9.0–9.2
Glaze	VITA AKZENT^®^ Plus Glaze	VITA Zahnfabrik, Bad Säckingen, Germany	63670	SiO_2_ (52–68%), Na_2_O (4–6%), Al_2_O_3_ (6–7%), K_2_O (3–4%), CaO (4–5%), B_2_O_3_ (9–11%), SnO_2_ (3–4%), ZrO_2_ (2–4%)	-

**Table 2:** Table with details of the regeneration firing and veneering porcelain.

	Pre-Drying		Heating Rate (°C/min)	Firing Temperature (°C)	Holding Time (min)
	Temperature (°C)	Time (min)			
Regeneration firing	500	0	100	1000	15
VITAVM9^®^ CORRECTIVE	500	4	80	760	1
VITA AKZENT^®^ Plus	500	4	80	900	1

**Table 3:** Surface treatment protocol and group code.

Surface Treatment	Method	Equipment	Code
No treatment (Control)	No further treatment after sintering.	-	C
Grinding	Grinding with 150-grit silicon carbide (3M wet or dry abrasive sheet, 3M-ESPE, Saint Paul, MN, USA) for 20 s where the roughness was equal to a 90–106 μm diamond bur.	Nano 2000 Polishing Machine (Nano 2000, Pace Technologies, Tuscon, AZ, USA)	GR
Airborne-particle abrasion with Al_2_O_3_	Specimens were abraded with 110 μm Al_2_O_3_ particles under a pressure of 0.4 MPa for 10 s at a distance of 10 mm.	Airborne-particle abrasion device (Basic Quattro, Renfert, Hilzingen, Germany)	AB
Glazing	VITA AKZENT Plus Glaze was applied on the zirconia surface by brush. One-hundred-micron plastic tape (3M, 3M-ESPE, Saint Paul, MN, USA) was used to control the thickness of the glaze material. The glazed zirconia specimens were fired at the temperature recommended by the manufacturer.	Porcelain furnace (Ivoclar Vivadent Programat 300, Ivoclar Vivadent, Schaan, Liechtenstein)	GL

**Table 4:** Rietveld analysis of the phase composition of zirconia types after surface treatment.

Type of Zirconia	Surface Treatment	c-ZrO_2_ ^a^ (wt%)	t-ZrO_2_ ^b^ (wt%)	m-ZrO_2_ ^c^ (wt%)	r-ZrO_2_ ^d^ (wt%)	GoF ^e^
3Y-TZP	C	43.35	52.13	0.60	3.93	1.32
	GR	32.14	35.93	2.80	29.13	1.13
	AB	38.62	32.78	3.93	24.67	1.16
4Y-PSZ	C	65.13	31.08	2.06	1.73	1.30
	GR	37.35	32.15	3.30	27.20	1.19
	AB	37.54	31.22	3.49	27.75	1.07
5Y-PSZ	C	82.84	16.42	0.60	0.13	1.67
	GR	47.92	27.04	3.09	21.94	1.26
	AB	35.82	29.97	3.87	30.33	1.12

^a^ Cubic zirconia, ^b^ tetragonal zirconia, ^c^ monoclinic zirconia, ^d^ rhombohedral zirconia, and ^e^ goodness of fit.

**Table 5:** Surface roughness measurements of zirconia types after surface treatment.

Type of Zirconia	Surface Roughness (µm)			
	C	GR	AB	GL
3Y-TZP	0.3319	0.0610	0.3661	0.0241
4Y-PSZ	0.2984	0.1361	0.3777	0.0229
5Y-PSZ	0.2373	0.1131	0.3667	0.0247

**Table 6:** The two-way ANOVA of SBS.

Source	Type III Sum of Squares	df	Mean Square	F	Sig.
Corrected Model	944.027 ^a^	11	85.821	4.946	<0.001
Intercept	65,415.492	1	65,415.492	3770.098	<0.001
Types	347.619	2	173.810	10.017	<0.001
Surface	443.522	3	147.841	8.521	<0.001
Types * Surface	152.886	6	25.481	1.469	0.196
Error	1873.923	108	17.351		
Total	68,233.442	120			
Corrected Total	2817.950	119			

^a^ R Squared = 0.335 (Adjusted R Squared = 0.267).

**Table 7:** The mean SBS and SD (MPa) in each group and significant differences within factors.

Types of Zirconia	Surface Treatment				Mean SBS
	C	GR	AB	GL	
3Y-TZP	23.22 (5.22)	22.03 (4.82)	29.34 (5.82)	27.09 (4.44)	25.42 (5.73) ^a^
4Y-PSZ	19.77 (4.78)	23.30 (2.17)	24.76 (3.16)	25.67 (5.00)	23.38 (4.43) ^a,b^
5Y-PSZ	19.43 (3.44)	21.23 (3.52)	22.30 (2.07)	22.04 (3.62)	21.25 (3.30) ^b^
**Mean SBS**	20.81 (4.72) ^A^	22.19 (3.64) ^A,B^	25.46 (4.87) ^C^	24.93 (4.75) ^B,C^	

The same superscript letter means there is no statistically significant difference between types of surface treatment. Uppercase letters indicate statistical differences between surface treatment groups. Lowercase letters indicate statistical differences between zirconia groups.

## Data Availability

The data presented in this study are available from the corresponding author upon reasonable request.
